# Encapsulated Pterostilbene in pH-Sensitive Alginate Beads for LDL Oxidation Inhibition and Antioxidant Protection

**DOI:** 10.1021/acsomega.5c07990

**Published:** 2026-03-17

**Authors:** Rener Mateus Francisco Duarte, Jéssica Maria Pereira, Livia Maria Santos de Lima, Tarcísio Paiva Mendonça, Vinicius Prado Bittar, Maria Sol Peña Carrillo, Jeniffer Mclaine Duarte de Freitas, Ilza Fernanda Barboza Duarte Rodrigues, Johnnatan Duarte de Freitas, Irinaldo Diniz Basílio Júnior, Allisson Benatti Justino, Foued Salmen Espindola, Anielle Christine Almeida Silva

**Affiliations:** 1 Strategic Materials Laboratory, Institute of Physics, 28112 Federal University of Alagoas, Maceió 57072-900, AL, Brazil; 2 Biochemistry and Molecular Biology Laboratory, Institute of Biotechnology, Federal University of Uberlandia, Uberlandia 38408-100, Brazil; 3 Northeast Biotechnology Network (RENORBIO), Federal University of Alagoas, Maceio 57072-900, Brazil; 4 Drug Technology and Control Laboratory, Institute of Pharmaceutical Sciences, Federal University of Alagoas, Maceio 57072-900, Brazil; 5 Laboratory of Instrumental Analysis, Federal Institute of Alagoas, Maceio 57020-600, Brazil

## Abstract

Dietary intake of bioactive compounds from fruits and vegetables has been associated with a reduced risk of chronic diseases. Pterostilbene, a naturally occurring stilbenoid and dimethylated analogue of resveratrol, is abundant in blueberries and exhibits potent antioxidant and anti-inflammatory properties, highlighting its potential for cardiovascular applications. However, its clinical translation is limited by poor stability and low bioavailability. Here, pterostilbene was encapsulated in alginate-based microcapsules to protect the compound from premature degradation and enable controlled, pH-responsive release. The microcapsules were synthesized by ionic gelation and characterized by scanning electron microscopy (SEM) and Fourier transform infrared spectroscopy (FTIR). Encapsulation efficiency reached 95.6%. Antioxidant capacity was assessed in vitro using DPPH, ferric reducing antioxidant power (FRAP), and oxygen radical absorbance capacity (ORAC) assays. The protective effect against oxidative stress was further evaluated by measuring the inhibition of Cu^2+^-induced human low-density lipoprotein (LDL) oxidation, followed by lipid peroxidation analysis using the TBARS assay. The microcapsules remained stable for up to 5 h under acidic conditions and fully dissolved within 1 h 38 min at basic pH. Encapsulation significantly enhanced the antioxidant activity of pterostilbene and improved its ability to inhibit LDL oxidation, supporting the potential of alginate-based delivery systems for cardiovascular applications.

## Graphical abstract

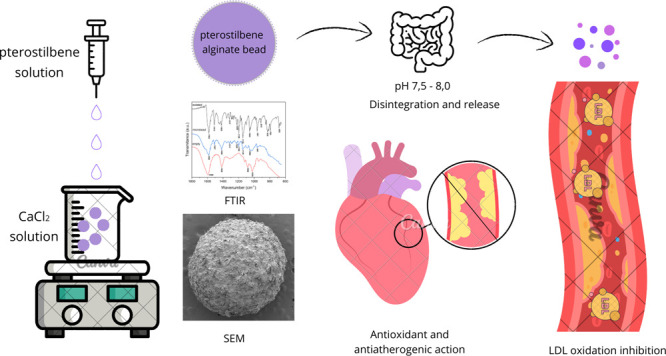

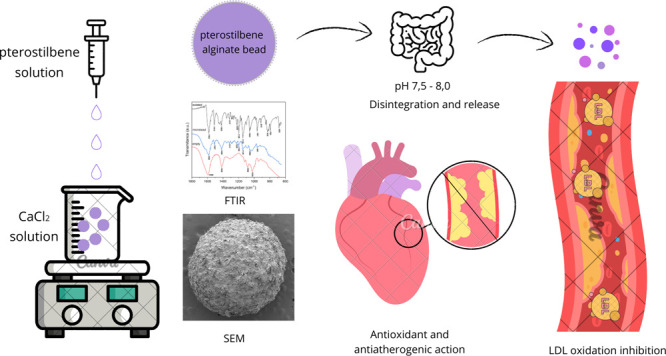

## 1 Introduction

Pterostilbene (PTR) is a naturally occurring polyphenol of the *trans*-stilbene class and a dimethylated analogue of resveratrol. Chemically defined as 3′,5′-dimethoxy-4-hydroxystilbene, PTR shares many of the biological activities attributed to resveratrol but frequently exhibits greater potency and improved pharmacological performance.^1–3^ One of PTR’s key advantages is its enhanced metabolic stability, allowing a higher fraction of the compound to be absorbed, reach its target sites, and remain unaltered for a longer duration compared to resveratrol.^4,5^

Naturally occurring compounds of this type are present in several plant species, particularly in grape varieties and blueberries,^6^ though it has also been identified in other plants. Its widespread presence in nature is associated with a potential protective role, acting as a natural antitoxic compound.^7^ Due to the presence of two methoxy groups in its structure, pterostilbene is more lipophilic than resveratrol, which enhances its intestinal permeability, cellular uptake, and overall stability.^8^

Encapsulation strategies, particularly those based on ionic gelation, have emerged as effective approaches to further improve the stability and bioavailability of bioactive polyphenols such as pterostilbene. Although flavonoids exhibit well-established antioxidant properties, their therapeutic application is often limited by susceptibility to environmental factors, including light, temperature, and oxygen.^9^ Encapsulation within polymeric matrices provides a protective microenvironment that preserves chemical integrity under adverse conditions and supports sustained bioactivity in biological systems.^10^ Importantly, ionic gelation–based encapsulation addresses a central limitation of oral flavonoid delivery, poor bioavailability resulting from premature degradation in the gastrointestinal tract, by facilitating the controlled delivery of the active compound to its target sites.^11^

Atherosclerosis is a pathological condition marked by arterial wall thickening, caused by chronic inflammatory responses triggered by lipid accumulation. This process can result in arterial occlusion, limiting blood flow and leading to tissue necrosis due to oxygen deprivation.^12^ Central to this process is oxidative stress, which arises when the generation of reactive oxygen species (ROS) exceeds endogenous antioxidant defenses. Excessive ROS promotes the oxidation of low-density lipoprotein (LDL), yielding oxidized LDL (ox-LDL), a key pro-inflammatory mediator that accelerates endothelial dysfunction, foam cell formation, and atherogenesis.^13,14^

Given the importance of oxidative stress and LDL oxidation in cardiovascular pathology, antioxidant-based strategies have attracted considerable interest as potential therapeutic approaches. Pterostilbene exhibits well-documented cardioprotective and antioxidant properties; however, its clinical translation is hindered by limited aqueous solubility, susceptibility to degradation, and rapid metabolic clearance.^15^ Encapsulation of pterostilbene within alginate-based matrices represents a promising strategy to overcome these limitations. Alginate is a biocompatible and biodegradable biopolymer that provides a protective microenvironment, shielding the encapsulated compound from premature degradation while enabling controlled and sustained release. This delivery approach enhances compound stability, prolongs antioxidant activity, and ultimately maximizes the therapeutic potential of pterostilbene in redox-driven cardiovascular disorders.

## 2 Materials and Methods

### 2.1 Preparation of Pterostilbene-Loaded Alginate Spherical Capsules

The spherical capsules were prepared using a modified ionic gelation method, as previously described in our patent BR102022010600, which exploits the electrostatic interaction between oppositely charged species to promote the formation of a stable three-dimensional polymeric matrix encapsulating the natural product. Briefly, sodium alginate (ALG) was employed as the natural biopolymer, and calcium chloride (CaCl_2_) as the cross-linking agent. The encapsulation formulation was prepared by dispersing 0.5 g of lyophilized pterostilbene (Uniflora, Brazil) into 5 mL of the 3% (w/v) sodium alginate solution under continuous magnetic stirring to ensure complete homogenization.

The cross-linking solution was prepared by dissolving CaCl_2_ in deionized water at a concentration of 3%. The alginate–pterostilbene mixture was then slowly added dropwise into the calcium solution using a syringe with a standardized nozzle diameter, allowing for the instantaneous formation of microspheres via ionic cross-linking. The resulting microspheres were gently collected by vacuum filtration, rinsed with distilled water to remove excess Ca^2+^ ions, and subsequently transferred to airtight containers. Samples were stored at 10 °C until further physicochemical and biological analyses. Blank microspheres (without pterostilbene) were prepared under the same conditions as controls. All experiments were carried out in triplicate at room temperature, and data are expressed as mean ± standard deviation.

### 2.2 High-Performance Liquid Chromatography-Electrospray Ionization-Tandem Mass Spectrometry (HPLC-ESI/MSn)

The HPLC-ESI/MSn method was used to identify the majority compounds present in the pterostilbene according to ref 16. The chromatograms and sequential mass spectra of the identified compounds can be found in the Supporting Information.

### 2.3 Spectrophotometric Characterization and Antioxidant Activity of Pterostilbene

The UV–Vis spectra of pure pterostilbene were recorded between 230 and 1000 nm using a spectrofluorimeter (EnSpire, PerkinElmer, USA), identifying the maximum absorption wavelength (λmax) between 290 and 300 nm. For this analysis, a serial 2-fold dilution was performed, beginning with an initial quantity of 5 mg and progressively reducing the amount by half at each step. A standard calibration curve was constructed at this wavelength using serial dilutions of pterostilbene from an initial stock solution of 2 mg/mL, yielding a linear regression equation (*A* = *aC* + *b*, *R* ^2^ > 0.96). Alginate microbeads were prepared by dispersing 0.25 g of pterostilbene in 8 mL of sodium alginate and dripping the mixture into 100 mL of 3% CaCl_2_ solution under magnetic stirring, forming approximately 80 spherical beads. After hardening and washing, the beads were dissolved in 1.5 mL of solvent for spectrophotometric analysis. Antioxidant activity was assessed using the DPPH radical scavenging assay. Different concentrations of pterostilbene and an extract obtained from one microbead were reacted with DPPH solution, and absorbance was measured at 517 nm after 30 min in the dark. Water was used as the negative control and quercetin 1% as the positive control. Results were expressed as the percentage of DPPH inhibition relative to the control.

### 2.4 Encapsulation Efficiency

For direct quantification, six alginate microbeads were dissolved in 1.5 mL of citrate buffer (0.55 mM) under vigorous stirring until complete gel disintegration. For indirect quantification, the supernatant obtained from the CaCl_2_ cross-linking medium was analyzed to determine the amount of unencapsulated (free) pterostilbene. This method provides a more reliable estimation of encapsulation efficiency by avoiding potential bias due to incomplete bead dissolution. Pterostilbene quantification was performed using UV–Vis spectrophotometry at λmax = 306 nm. A standard calibration curve was constructed over the concentration range of 2.0–0.001953 mg·mL^–1^, and all absorbance readings were corrected by subtracting the alginate blank (Abs = A_sample – A_blank). Concentrations were then calculated from the inverted regression equation (*y* = −0.4672*x* + 3.4721; *R* ^2^ = 0.9691).

The encapsulation efficiency (EE%) was calculated by the indirect method according to eq 1:

$$\mathrm{EE}\%=\frac{Ctotal-Cfree}{Ctotal}x100$$ 1

where *C*_total_ represents the initial amount of pterostilbene used for encapsulation and *C*_free_ corresponds to the concentration of nonencapsulated drug determined in the supernatant.

### 2.5 Scanning Electron Microscopy (SEM)

Morphological characterization of the capsules was conducted using a scanning electron microscope (SEM) (Tescan VEGA3). One capsule from each formulation was mounted on double-sided carbon conductive tape and coated with a thin layer of gold using a metallizer (Quorum Q150R ES) to enhance imaging for analysis.

### 2.6 Attenuated Total Reflection Fourier Transform Infrared (ATR-FTIR) Spectroscopy

The capsules were dried and pressed for analysis ATR-FTIR spectroscopy analysis. The analysis was performed using a Cary 660 spectrometer (Agilent, California) equipped with an attenuated total reflectance (ATR) accessory. Spectra were recorded to identify the chemical functional groups present in the formulations.

### 2.7 Acid and Basic pH Resistance Test

The dissolution behavior of the encapsulated beads was evaluated under two distinct pH conditions to simulate gastric and intestinal environments. For each condition, six beads were placed in a beaker containing 30 mL of dissolution medium and maintained at 37 °C under constant agitation at 100 rpm. The acidic condition was established using hydrochloric acid (pH 1.2), while the basic condition was adjusted to pH 8.0 using an appropriate buffer. The experiment was carried out for a total duration of 5 h in each medium. Bead integrity and dissolution were visually monitored throughout the assay to assess the stability of the formulation under different pH environments.

### 2.8 Antioxidant Activity

To determine the antioxidant capacity of the samples, we performed three different assays: ferric reducing antioxidant power (FRAP), 2,2-diphenyl-1-picrylhydrazyl (DPPH) antioxidant activity, and oxygen radical absorbance capacity (ORAC). In the case of the encapsulated formulation, the alginate microbeads were fully dissolved prior to analysis to ensure unbiased testing and complete availability of the encapsulated pterostilbene. The FRAP method evaluates the ability of the sample to reduce ferric ions, the ORAC assay measures the capacity to scavenge peroxyl radicals, and the DPPH assay challenges the sample to reduce the DPPH radical to its hydrazine form.

#### 2.8.1 FRAP Assay

The samples were mixed with the FRAP reagent (composed of 300 mM sodium acetate buffer pH 3.6, 2,4,6-tri-(2-pyridyl)-s-triazide (TPTZ), and 20 mM ferric chloride in a 10:1:1 ratio, respectively) and incubated for 6 min at 37 °C. After incubation, absorbance was measured at 593 nm using a microplate reader (EnSpire, PerkinElmer, USA). A standard curve for 6-hydroxy-2,5,7,8-tetramethylchroman-2-carboxylic acid (Trolox) was constructed. Quercetin was used as a positive control and sodium acetate buffer was used as a negative control. The results were expressed as μmol of trolox (eq g^–1^).^17,18^

#### 2.8.2 ORAC Assay

The samples were incubated with 0.085 mM fluorescein and 153 mM of 2,2′-azobis­(2-amidinopropane) dihydrochloride (AAPH); both reagents were diluted in 75 mM phosphate buffer (pH 7.4). Fluorescence was measured at 485 nm excitation and 528 nm emission for 90 min at 37 °C in a microplate reader (EnSpire, PerkinElmer, USA). An analytical standard curve of Trolox was constructed to measure the antioxidant capacity of the samples in the assay. Phosphate buffer was used as a negative control, and quercetin was used as a positive control. The results were expressed as μmol of Trolox (eq g^–1^).^19^

#### 2.8.3 DPPH Assay

The DPPH assay was conducted following the method described before,^20^ with modifications.^21^ The samples were incubated with 60 mM DPPH radical (diluted in methanol) for 30 min at 37 °C in the absence of light. Subsequently, absorbance was measured at 517 nm in a microplate reader (EnSpire, PerkinElmer, USA). The DPPH scavenging capacity of the samples was determined using the following formula: DPPH (%) = [(Abs DPPH – Abs sample)/(Abs DPPH – A blank)] × 100, where Abs DPPH refers to the absorbance of DPPH solution, Abs sample refers to the absorbance of the sample/positive control mixed with DPPH solution, and Abs blank refers to the absorbance of the sample mixed with only methanol. Methanol was used as a negative control and quercetin was used as a positive control.

### 2.9 Isolation, Oxidation, and Peroxidation of Low-Density Lipoprotein (LDL)

#### 2.9.1 Isolation and Purification of Human LDL

Low-density lipoprotein (LDL) was isolated following the method already described,^22^ from peripheral blood obtained from healthy, nonsmoking adult volunteers, in accordance with ethical approval granted by the Human Research Ethics Committee of the UNA University Center, Uberlândia, Brazil (protocol no. 5.671.038; in compliance with Resolution CNS 466/12 of the Brazilian National Health Council). Blood samples were collected in EDTA-containing vacutainer tubes (10%) and centrifuged at 800 × *g* for 10 min at 4 °C to separate the plasma. To inhibit proteolysis and oxidation, plasma was supplemented with a protease and antioxidant inhibitor cocktail comprising: aprotinin (5 μL/mL plasma), benzamidine (2 mM, 5 μL/mL), phenylmethylsulfonyl fluoride (PMSF, 0.5 mM, 0.5 μL/mL), chloramphenicol (0.25%, 0.5 μL/mL), and a preservative solution containing 5% sodium azide, 8% EDTA, and 0.1% chloramphenicol (10 μL/mL). The plasma density was then adjusted to 1.21 g/mL using potassium bromide. LDL was isolated by sequential ultracentrifugation using a Sorvall WX 90+ ultracentrifuge (Thermo Fisher Scientific) at 53,000 × *g* for 2.5 h at 4 °C. The distinct orange LDL-containing band was carefully collected with a syringe and subjected to dialysis against phosphate-buffered saline (PBS 1×, pH 7.4) for 24 h at 4 °C, with buffer changes every 6 h to remove residual salts and reagents. Following purification, LDL samples were stored at 4 °C protected from light until use. Total protein content was determined using a modified Bradford assay^23^ with modifications.

#### 2.9.2 Assessment of Copper-Induced Oxidative Modification of LDL

The oxidative modification of low-density lipoprotein (LDL) was initiated by the addition of 5 μM copper­(II) sulfate, and the reaction kinetics were monitored for 2 h at 37 °C. Absorbance measurements were recorded at 2 min intervals at 234 nm, corresponding to the formation of conjugated dienes,^24^ using a microplate reader (EnSpire, PerkinElmer, USA). This approach enabled the determination of the lag phase, defined as the intersection between the initial low-rate and subsequent high-rate phases of diene formation. Experimental samples were tested at a final concentration of 1 μg/mL, and phosphate-buffered saline (PBS) served as a negative control. Following the kinetic analysis, aliquots were collected directly from the microplate wells, and lipid peroxidation levels were quantified using the thiobarbituric acid reactive substances (TBARS) assay to estimate malondialdehyde (MDA) content.

#### 2.9.3 Quantification of Lipid Peroxidation via TBARS Assay

Lipid peroxidation was quantified by assessing malondialdehyde (MDA) levels through a thiobarbituric acid reactive substances (TBARS) assay.^25^ Briefly, samples were incubated with 10% (w/v) trichloroacetic acid (TCA) and 0.67% (w/v) thiobarbituric acid (TBA) for 2 h in a water bath at 100 °C. Following thermal incubation, 400 μL of *n*-butanol was added to each sample to extract the MDA-TBA adduct. The mixtures were then centrifuged at 3000 × *g* for 10 min, and the organic phase was collected for analysis. Fluorescence intensity was measured at 532 nm using an EnSpire multimode plate reader (PerkinElmer, USA). The MDA concentration was calculated using a standard curve and normalized to total protein content, with results expressed as nanomoles of MDA per milligram of protein (nmol/mg protein).

## 3 Results

### 3.1 Purity Analysis by HPLC-ESI-MS^n^

The analysis using high-performance liquid chromatography coupled with electrospray ionization tandem mass spectrometry (HPLC-ESI-MS^n^) was performed to assess the purity of the encapsulated pterostilbene. The resulting chromatogram (Table 1) exhibited the exclusive presence of the target compound, with no detectable impurities, degradation products, or contaminants. These findings confirm the chemical purity and integrity of the encapsulated pterostilbene. Additional chromatographic data are provided in the Supporting Information (see Figure S1).

**Table 1:** Compound Identified in the Pterostilbene Samples by HPLC-ESI-MS/MS (Negative and Positive Modes)

*N°* compound	Tentative identity	Retention time (min)	Formula	Mass calculated	*m*/*z* observed	Error (ppm)	*m*/*z* of fragments of [M–H]^−^	References
1	Pterostilbene	14.275	C_16_H_15_O_3_^–^ [M–H]^−^	255.1027 [M–H]^−^	255.1027 [M–H]^−^	0.0	-	^200^

### 3.2 Spectrophotometric Characterization and Antioxidant Activity of Pterostilbene

The UV–Vis absorption spectra of pterostilbene at three different concentrations and the corresponding blank solution are shown in Figure 1. All spectra exhibited a characteristic absorption band between 290 and 300 nm, consistent with the π–π transition of the stilbene aromatic system, confirming the identity and spectral purity of the compound. Increasing concentrations produced proportional increases in absorbance intensity, validating the linearity of the response and the suitability of this wavelength for quantitative analysis (Figure 1A). The standard calibration curve constructed at 200–700 nm showed excellent linearity (*R* ^2^ 0.9691), and this equation was used for the quantification of pterostilbene in the encapsulated and nonencapsulated samples (Figure 1B). The antioxidant activity of pterostilbene was then assessed using the DPPH radical scavenging assay. As shown in (Figure 1C), the antioxidant effect was concentration-dependent, with higher concentrations of pterostilbene resulting in greater inhibition of the DPPH radical. When the extract obtained from a single alginate microbead was evaluated under the same conditions, it also exhibited measurable antioxidant activity, demonstrating that the encapsulated compound retains its radical scavenging potential after entrapment within the alginate matrix.

**Figure 1:**
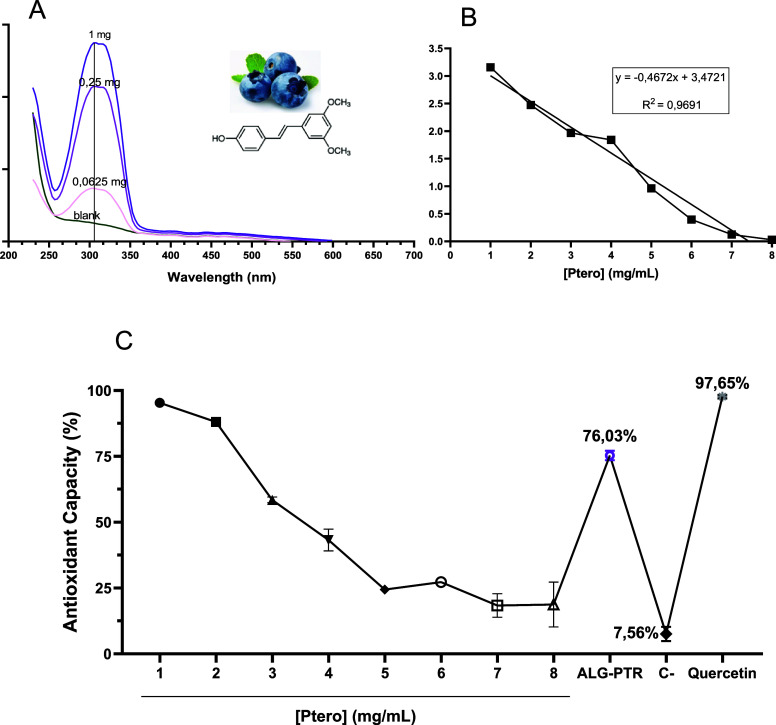
Spectrophotometric and antioxidant characterization of pterostilbene. (a) UV–Vis absorption spectra of pterostilbene at varying concentrations, compared with blank. (b) Calibration curve (absorbance vs concentration), prepared from a serial dilution with an initial concentration of 5 mg/mL. (c) DPPH radical scavenging activity of free pterostilbene and pterostilbene released from one dissolved bead; water was used as the negative control and 1% quercetin as the positive control.

### 3.3 Encapsulation Efficiency

The alginate microbeads exhibited an average diameter of 0.30 cm, corresponding to an estimated mean volume of 14 μL per unit. Based on the initial pterostilbene concentration used in the precursor solution (31.25 mg mL^–1^), the theoretical drug loading was calculated as 0.44 mg of pterostilbene per microbead. Encapsulation efficiency (EE%) was first assessed by the indirect method, in which the mass of nonencapsulated pterostilbene remaining in the supernatant after gelation was quantified. This approach indicated minimal drug loss during bead formation and corresponded to EE values above 90%, confirming that alginate effectively retained pterostilbene during ionic cross-linking. To complement this analysis, a direct quantification method was performed by dissolving individual microbeads and measuring the released pterostilbene. This method yielded a mean loading of 0.55 mg per microbead, which is moderately higher than the theoretical value. Additional physicochemical parameters are summarized in the Supporting Information (Table S1).

### 3.4 Morphological Analysis

Morphological analyses using scanning electron microscopy (SEM) revealed structural and surface differences between the ALG (empty spheres) and those containing pterostilbene (Figure 2). Both types exhibited well-preserved surfaces, with indications of porosity and no visible cracks. Formulation A1-A3 displayed an irregular yet smooth surface, characteristic of empty particles, because of the drying process. This phenomenon occurs due to water loss, which weakens the gel matrix structure, leading to shrinkage and partial surface collapse of the spheres. In contrast, when comparing formulations, A and B, it was observed that the pterostilbene extract acted as a structuring agent, aiding in the preservation of the spherical shape of the particles. Formulation B demonstrated greater resistance to drying, resulting in a more uniform granular surface without evidence of cracks. These findings reinforce the effectiveness of the encapsulation process and the stability of the employed technique.

**Figure 2:**
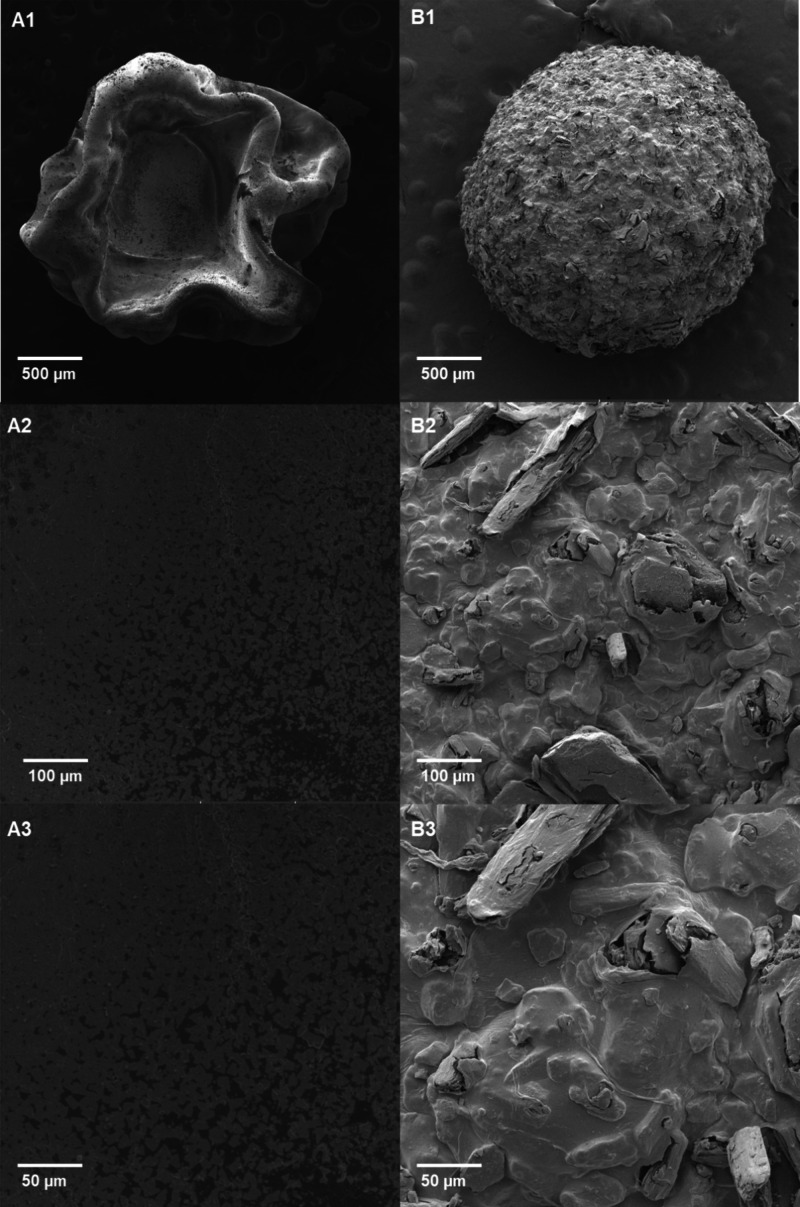
Scanning electron microscopy (SEM) images of alginate microbeads samples. Representative SEM micrographs of alginate beads (A1–A3) and alginate–pterostilbene beads (B1–B3) acquired at scale bars of 500, 100, and 50 μm, respectively.

### 3.5 FTIR Analysis of Isolated and Encapsulated Samples

Fourier transform infrared spectroscopy (FTIR) analysis was conducted to assess the chemical interactions between isolated pterostilbene, empty microspheres, and microspheres containing pterostilbene (Figure 3). The spectrum of PTR exhibited characteristic bands associated with the compound’s functional vibrations, such as the band at 1582 cm^–1^, attributed to aromatic ring stretching vibrations, and the band at 1224 cm^–1^, which can be related to the stretching vibrations of C–O groups bonded to aromatic structures.^26^ In the ALG, bands at 1595 and 1420 cm^–1^ were observed, corresponding to the polymer used in the encapsulation process, thereby confirming its chemical composition. ALG-PTR (microspheres loaded with pterostilbene) displayed overlapping bands from both the empty microspheres and the isolated compound, confirming the successful incorporation of pterostilbene into the encapsulation system without significant chemical interactions that could alter the molecular structure of the compound. These findings demonstrate that the encapsulation process preserved the chemical integrity of pterostilbene while maintaining the structural characteristics of the microspheres.

**Figure 3:**
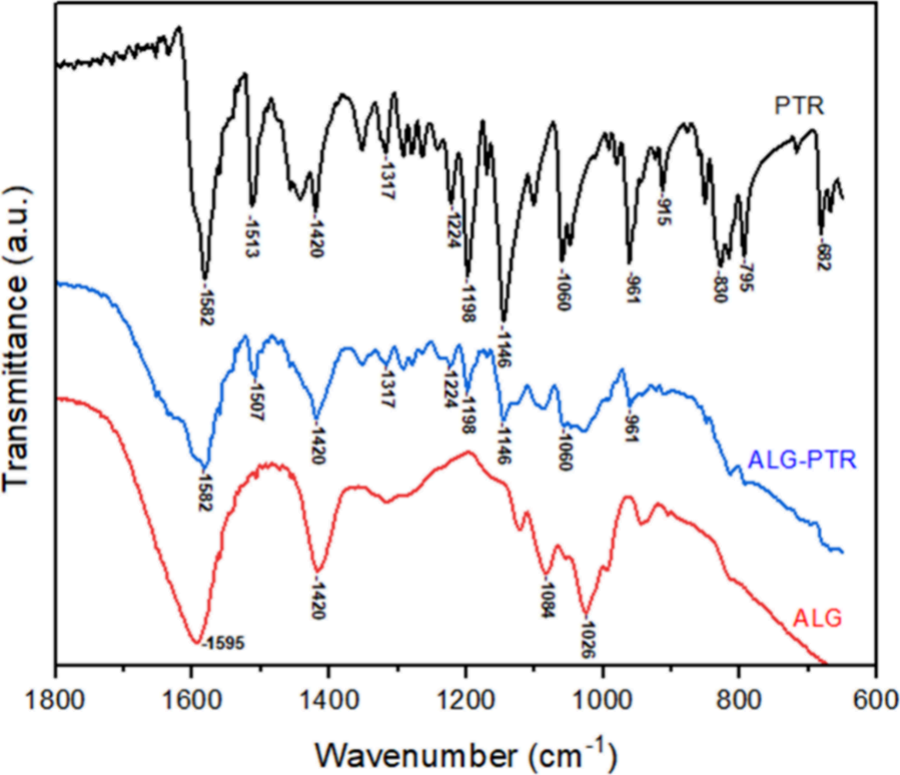
FTIR spectra of pterostilbene (PTR), pterostilbene-loaded alginate (ALG-PTR), and unloaded alginate beads (ALG), acquired using attenuated total reflectance (ATR) mode.

### 3.6 Acid and Alkaline pH Resistance Test

Under acidic pH conditions, the microbeads exhibited an immediate color change upon contact with HCl, developing a bronze-like appearance. Over time, there was a slight reduction in their size; however, they remained intact and resistant throughout the 5 h agitation period at pH 1.2. In contrast, under basic pH conditions, the microbeads showed no immediate color change. After approximately 1 h, they began to develop a whitish and hydrated appearance, eventually dissolving completely within approximately 1 h and 38 min (Table 2).

**Table 2:** Results of the pH Resistance Assay of Pterostilbene-Loaded Beads under Different pH Conditions

pH	time (h)
1.2	5 h
8.6	1 h 38 min

### 3.7 Assessment of Antioxidant Activity

In Figure 4, the DPPH assay revealed that the empty capsule showed no significant difference compared to the negative control (methanol), confirming the absence of antioxidant activity in the polymer and supporting the findings of other assays, which highlight the antioxidant inertness of the encapsulation material. In contrast, the ALG-PTR exhibited exceptionally high antioxidant activity (98.9%, *p* < 0.0001), demonstrating the effectiveness of the encapsulation process in preserving and enhancing the compound’s antioxidant functionality. In the FRAP assay, the pterostilbene-loaded microcapsule exhibited significantly higher antioxidant activity (*p* < 0.0001) compared to the empty capsule, which demonstrated a high capacity to reduce ferric ions, indicating that the encapsulation process fully preserved the antioxidant functionality of pterostilbene. In the ORAC assay, the ALG-PTR exhibited the highest antioxidant capacity among the tested groups (*p* < 0.0001), significantly surpassing both the ALG. These results confirm that the encapsulation process not only maintains but also enhances the bioactivity of the antioxidant compound.

**Figure 4:**
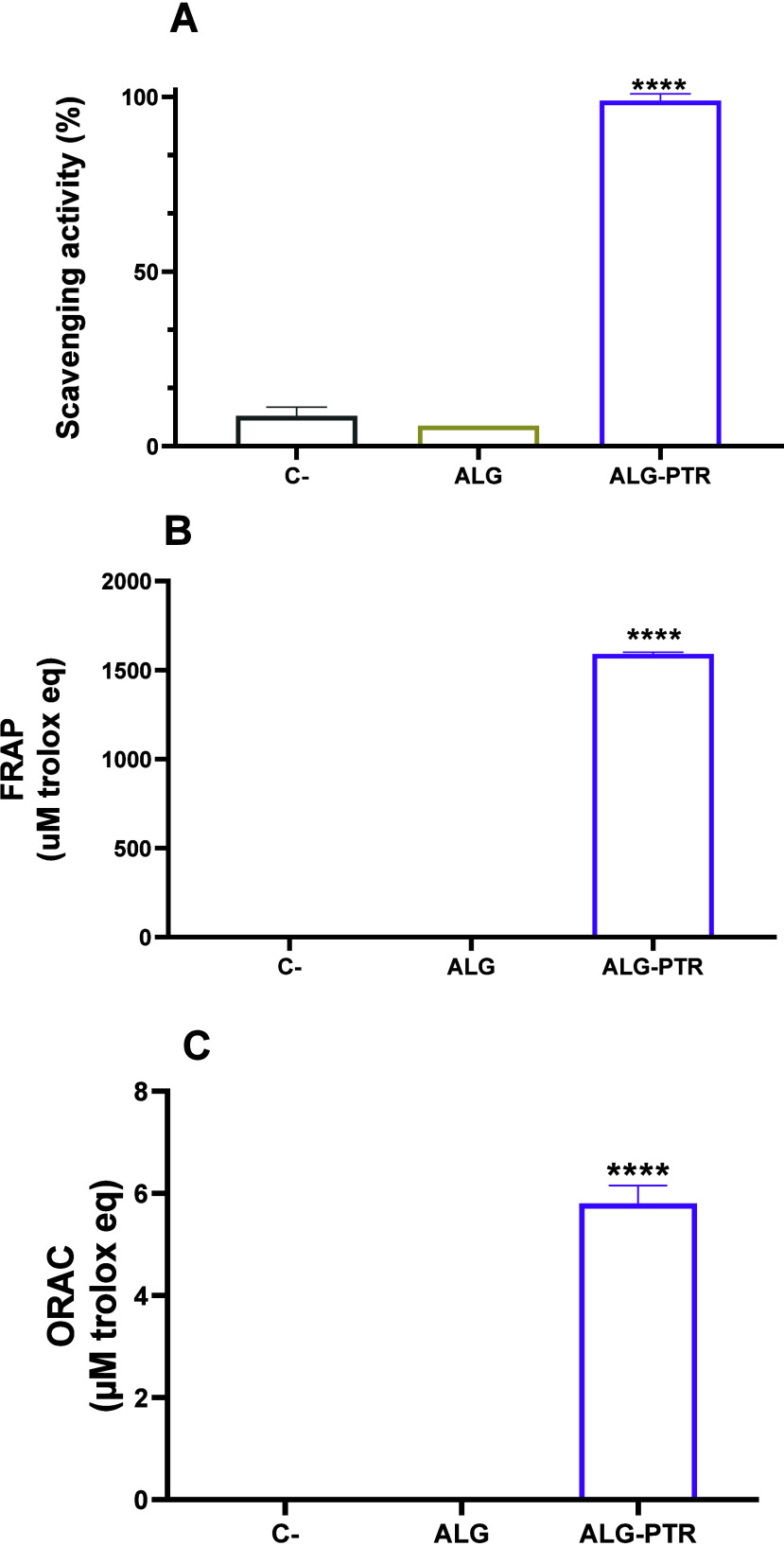
In vitro antioxidant capacity analysis. Antioxidant activity assessed by DPPH (A), FRAP (B), and ORAC (C) assays. Data are presented as mean ± s.d. FRAP and ORAC results are expressed as μM Trolox equivalents per mg, whereas DPPH results are expressed as percentage antioxidant activity. Methanol was used as the negative control. Statistical significance is indicated by asterisks (*p < 0.05).

### 3.8 Inhibition of LDL Oxidation and Peroxidation Levels

#### 3.8.1 LDL Oxidation Inhibition Activity

The LDL-ox group, treated with copper­(II) sulfate, exhibited a marked increase in lipid peroxidation, confirming the efficacy of the protocol in promoting oxidative modification of LDL. In contrast, the nonoxidized LDL control (without Cu^2+^) showed negligible levels of oxidation (*p* < 0.0001), reinforcing the specificity and reliability of the assay. PTR and encapsulated PTR significantly inhibited LDL oxidation when compared to the oxidized control (*p* < 0.0001 for both), demonstrating pronounced antioxidant activity. Notably, the extent of protection afforded by pterostilbene, particularly in its encapsulated formulation, was comparable to that of the reference antioxidant quercetin (*p* < 0.0001). These results underscore the potential of pterostilbene as a potent inhibitor of LDL oxidation, with the encapsulated form showing enhanced or at least equivalent efficacy, possibly due to improved stability or bioavailability conferred by the delivery system (Figure 5A).

**Figure 5:**
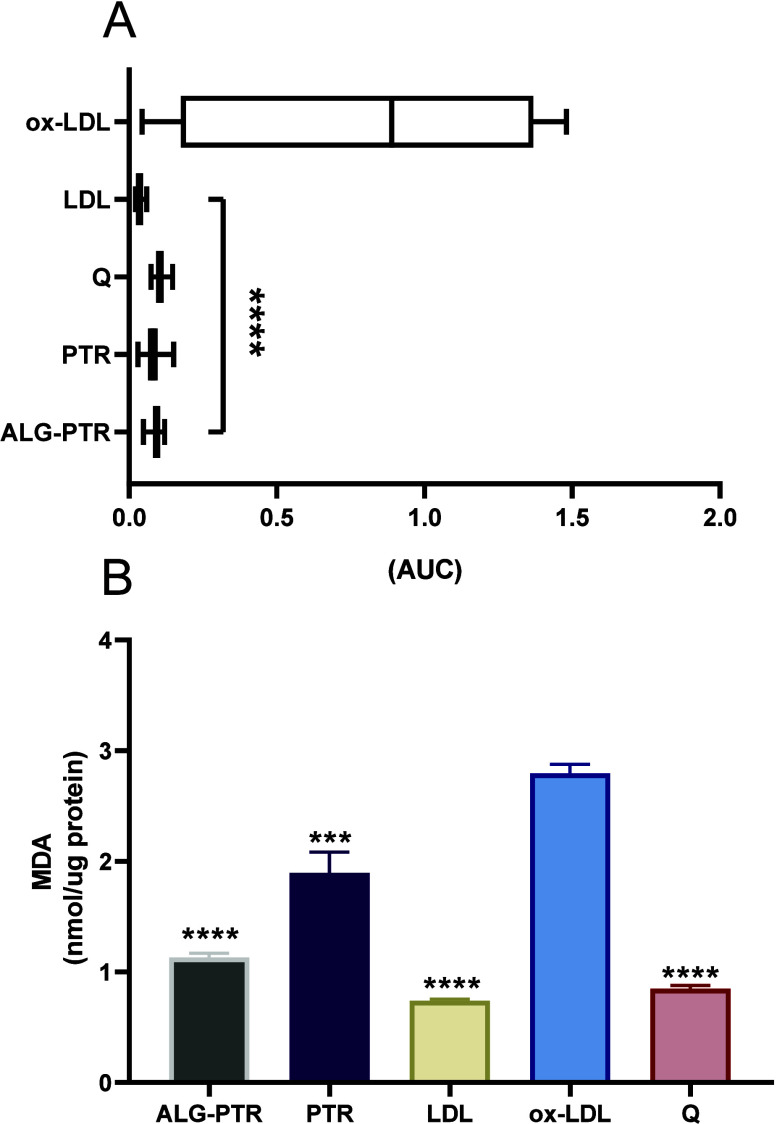
In vitro analysis of human low-density lipoprotein (LDL) oxidation. Human LDL was oxidized with Cu^2+^ and cotreated with quercetin or pterostilbene, either in isolated form or encapsulated in beads. LDL oxidation was quantified as the area under the curve (AUC) (A), and lipid peroxidation was assessed by malondialdehyde (MDA) levels (B). Data are presented as mean ± s.d. and expressed as AUC and μmol MDA per mg protein, respectively. All samples were tested at a concentration of 1 μg mL^–1^. ox-LDL, oxidized low-density lipoprotein. Quercetin was used as the positive control. Statistical significance is indicated by asterisks (*p < 0.05).

#### 3.8.2 Evaluation of Lipid Peroxidation

To confirm the occurrence of oxidative damage to LDL, lipid peroxidation was quantified via the TBARS assay by measuring malondialdehyde (MDA) formation. Treatment with PTR significantly reduced MDA levels compared to the oxidized LDL control (*p* = 0.0006), indicating a protective antioxidant effect. Notably, the encapsulated form of PTR demonstrated even greater efficacy, leading to a more pronounced decrease in lipid peroxidation (*p* < 0.0001). The reduction in MDA levels observed with both pterostilbene formulations was statistically comparable to those seen with the positive control, quercetin (*p* < 0.0001), and the nonoxidized LDL group (negative control; *p* < 0.0001). These results further reinforce the antioxidant potential of pterostilbene, particularly when encapsulated, in preventing oxidative modification of LDL particles (Figure 5B).

## 4 Discussion

To enhance the stability and biological performance of pterostilbene (PTR), we employed ionic gelation to produce calcium–alginate microbeads under mild and fully aqueous conditions. This encapsulation strategy was selected due to the known susceptibility of pterostilbene and other phenolic antioxidants to oxidation, photodegradation, and loss of activity in physiological environments.^27^ Alginate provides a biocompatible, biodegradable, and chemically gentle matrix that not only favors the entrapment of hydrophobic molecules but also protects them from premature degradation, thereby preserving their functional properties.^28,29^

Within this framework, ionic gelation proved to be a robust and reproducible method, enabling the incorporation of therapeutically relevant amounts of PTR with an encapsulation efficiency of 95.6%. Such high retention indicates a strong affinity between the phenolic compound and the alginate network, suggesting that the cross-linking process maintains drug integrity while supporting efficient loading for potential biomedical applications.

The preservation of PTR within the matrix is particularly important given that oxidative stress and abundant reactive oxygen species (ROS), key features of the atherosclerotic microenvironment, readily degrade free polyphenols, diminishing their therapeutic potential.^30,31^ UV–Vis and fluorescence measurements demonstrated the maintenance of PTR’s characteristic spectral features after encapsulation, while FTIR data confirmed noncovalent interactions between the molecule and the alginate network.

These findings indicate successful incorporation without chemical modification of the active compound and highlight the relevance of encapsulation for stabilizing PTR prior to antioxidant and LDL oxidation assays.^32,33^ Morphological evaluation by SEM revealed uniform spherical particles with smooth surfaces, indicating effective ionic cross-linking and homogeneous polymeric distribution. These structural characteristics are directly related to controlled release behavior and to the protection of the encapsulated antioxidant against environmental stressors.^34^

The pterostilbene-loaded microbeads retained substantial antioxidant capacity, as confirmed by DPPH, ORAC, and FRAP assays. Furthermore, the encapsulated compound effectively inhibited LDL oxidation, highlighting its potential to mitigate one of the earliest and most critical events in atherogenesis. This retention of biological activity after encapsulation underscores the efficiency of the alginate matrix in protecting and gradually releasing the compound in a bioactive form.^35,36^

Phenolic compounds are primarily absorbed in the small intestine after enzymatic hydrolysis into aglycones. However, glycosylated forms that resist digestion can reach the colon, where they are metabolized by the gut microbiota into bioactive derivatives that may be absorbed locally. Therefore, designing a delivery system that enables the release of phenolic glycosides in the large intestine is advantageous to exploit microbial biotransformation and enhance systemic bioavailability.^37,38^ Starch-based wall materials employed to encapsulate undergo degradation in the oral cavity, primarily due to the activity of α-amylase. Protein-based delivery systems are hydrolyzed in the stomach, exposed to acidic pH and the enzyme pepsin. Lipid-based particles typically release their core substances in the small intestine.^39^

A complementary direct quantification was performed by dissolving individual beads and measuring the released pterostilbene. This method yielded values higher than the theoretical maximum, a behavior frequently observed in manually produced alginate systems. The apparent overestimation likely reflects a combination of factors, including surface encrustation of pterostilbene during droplet formation, slight bead-to-bead volume heterogeneity, and more complete drug extraction upon dissolution. In addition, hydrophobic interactions between pterostilbene and the alginate network may enhance retention at both the surface and inner matrix, contributing to the higher measured loading.

The physicochemical characteristics of the substances employed in the development of encapsulants, along with their digestibility in the gastrointestinal tract and the localized bioactive action of the ingredient, are crucial factors. The microspheres exhibited robust stability in acidic pH, rendering them resistant during the transient gastric phase in the presence of hydrochloric acid. Upon reaching a basic pH of approximately 8.5 within a few hours, the polymeric matrix disintegrates, releasing the content into the highly absorptive tissue.

To ensure capsule integrity in the stomach and their release in the intestine, strategies such as the use of 3% alginate, providing enhanced resistance, and cross-linking with Ca^2+^, which improved stability in acidic conditions, were employed.^40,41^ The tests demonstrated that the capsules remained intact at pH 1.2, simulating the gastric environment, and fully dissolved at pH 8, confirming their intestinal release. This formulation represents a promising approach for the controlled oral delivery of compounds sensitive to gastric acidity.

Another crucial aspect is the safety of the microcapsule, as it must not induce toxicity in the organism, particularly in the gastrointestinal tissue. The safety of materials used in the encapsulation process is of paramount importance. A very limited number of coatings and excipient materials have been approved for food use. In some encapsulation methods, residues of nonfood solvents and detergents can pose health issues. The use of alginate has shown significant safety in these regards, being biocompatible depending on its composition and purity, as well as the content of M and G block.^42,43^

The antioxidant assays demonstrated that the incorporation of pterostilbene into alginate microbeads effectively preserved its redox capacity. In particular, the ORAC assay highlighted the sustained antioxidant potential of the loaded microbeads, confirming that the activity observed originates from the presence of the encapsulated compound. In contrast, empty alginate beads exhibited negligible activity, reinforcing that the protective matrix alone does not contribute significantly to radical scavenging.^44^ Complementary results from FRAP and DPPH assays further supported these results, demonstrating that the pterostilbene-loaded microbeads efficiently reduced iron ions and neutralized free radicals through distinct mechanisms of action.

Previous studies have reported that pterostilbene protects vascular endothelial cells against oxidized low-density lipoprotein (ox-LDL) by inducing apoptosis in these cells. This suggests that pterostilbene may serve as a potential natural antiapoptotic agent for atherosclerosis treatment.^45^ Additionally, pterostilbene has the potential to act as an antiproliferative agent for the treatment of atherosclerosis and angioplasty restenosis^46,47^ and neurological dysfunction associations.^48^

Furthermore, other studies have demonstrated its protective role in the cardiovascular system, attributed to its ability to influence various pathways.^49–51^ The administration of PTR facilitated the rehabilitation of glutathione metabolism and restored redox homeostasis in the right ventricle of monocrotaline-treated rats. At higher doses, PTR attenuated lipoperoxidation and while increasing the levels of sarcoplasmic reticulum calcium ATPase in the right ventricles of afflicted rodents.^52^ The endogenous antioxidant response of the vascular system is responsible for exerting a protective effect by mitigating oxidative damage. However, the antioxidant capacity may become depleted due to increased and chronic exposure to ROS, creating an imbalance between oxidative and antioxidative activities. Therefore, a prolonged release of antioxidant metabolites is highly desirable^8,53^

Scanning electron microscopy (SEM) analysis confirmed the successful encapsulation by revealing that the pterostilbene-containing formulation maintained a well-defined surface structure, with a shape consistent with a capsule or sphere. The absence of cracks or openings is crucial for reducing the permeability of the active compound, ensuring greater protection and retention of bioactive components. The morphological patterns observed in this study, including the formation of uniform, spherical structures with intact surfaces, are consistent with previous reports using the same ionic gelation technique, further supporting the reliability of the encapsulation method.^54^

In addition to morphological characterization, the antioxidant activity of the encapsulated formulations further supports the efficiency of the employed technique. The pterostilbene-loaded spheres exhibited significant antioxidant activity, whereas the empty capsules showed no antioxidant effect, confirming that the active substance was effectively incorporated and protected within the polymeric matrix. Encapsulation within alginate microspheres likely protects pterostilbene from early degradation and auto-oxidation, preserving its redox-active structure prior to interaction with LDL particles. Moreover, the hydrophilic nature of alginate may enhance the dispersibility of pterostilbene in aqueous environments, improving its accessibility to oxidizing agents and target substrates.^55^

Similar findings have been reported with other polyphenols such as resveratrol and curcumin, where encapsulation in biopolymers or lipid-based systems improved stability, solubility, and antioxidant performance.^56–58^ Alginate-based matrices have demonstrated efficacy in sustaining the release of bioactive and prolonging antioxidant activity over time.^59^ Such properties are particularly relevant for the development of nutraceuticals or drug delivery systems aimed at cardiovascular protection through sustained antioxidant action.^60,61^ This sustained release profile may contribute to continuous inhibition of lipid peroxidation throughout the oxidative process, reducing malondialdehyde accumulation and delaying the propagation of reactive species.

Despite the promising results obtained in vitro, further investigations are warranted to fully translate these findings into practical applications. Future studies should evaluate the stability and release profile of pterostilbene-loaded microbeads in gastrointestinal-like conditions and assess their bioavailability and metabolic fate in vivo. Additionally, exploring the potential synergistic effects of coencapsulated bioactive compounds or combining encapsulation with other therapeutic strategies could further enhance cardiovascular protection. Overall, the data suggests that the methodology employed in the generation of alginate microspheres can preserve the stability and antioxidant activity of pterostilbene, indicating its potential usefulness in future applications requiring the mitigation of reactive species in oxidative environments.

## 5 Conclusions

Alginate-based microencapsulation effectively preserved the antioxidant activity of pterostilbene. Notably, the encapsulated compound significantly inhibited LDL oxidation, likely due to its enhanced antioxidant stability. This strategy not only extends the functional lifespan of pterostilbene but also broadens its potential for use in cardiovascular-related applications. Furthermore, the pH-responsive behavior of the alginate capsules allows for protection in acidic gastric conditions and targeted release under intestinal pH, supporting efficient absorption and site-specific delivery.

## Supplementary Material



## Notes

(Figure S1) Chromatograms of pterostilbene extract samples by HPLC-ESI-MS/MS; (Table S1) physicochemical parameters and encapsulation characteristics of alginate–pterostilbene microbeads (PDF)

## References

[1] Kim H, Seo K. H., Yokoyama W (2020). Chemistry of Pterostilbene and Its
Metabolic Effects. J. Agric. Food Chem..

[2] Hsia C. W., Huang W. C., Yang C. H., Hsia C. H., Jayakumar T., Bhavan P. S. (2021). Comparison of the potency
of pterostilbene
with nf-κb inhibitors in platelet activation: Mutual activation
by akt-nf-κb signaling in human platelets. Appl. Sci..

[3] Tian R., Miao L., Cheang W. S. (2024). Effects
of Pterostilbene on Cardiovascular
Health and Disease. Curr. Issues Mol. Biol..

[4] Kapetanovic I. M., Muzzio M., Huang Z., Thompson T. N., McCormick D. L. (2011). Pharmacokinetics,
oral bioavailability, and metabolic profile of resveratrol and its
dimethylether analog, pterostilbene, in rats. Cancer Chemother Pharmacol..

[5] Wang P., Sang S. (2018). Metabolism and pharmacokinetics
of resveratrol and pterostilbene. BioFactors..

[6] Rimando A.
M., Kalt W., Magee J. B., Dewey J., Ballington J. R. (2004). Resveratrol,
pterostilbene, and piceatannol in Vaccinium berries. J. Agric. Food Chem..

[7] Badiali C., Beccaccioli M., Sciubba F., Chronopoulou L., Petruccelli V., Palocci C. (2024). Pterostilbene-loaded
PLGA nanoparticles alter phenylpropanoid and oxylipin metabolism in
Solanum lycopersicum L. leaves. Sci. Rep..

[8] Nagarajan S., Mohandas S., Ganesan K., Xu B., Ramkumar K. M. (2022). New Insights
into Dietary Pterostilbene: Sources, Metabolism, and Health Promotion
Effects. Molecules.

[9] Estrela J. M., Ortega A., Mena S., Rodriguez M. L., Asensi M. (2013). Pterostilbene: Biomedical applications. Crit Rev. Clin Lab Sci. maio de.

[10] Řepka D., Kurillová A., Murtaja Y., Lapčík L. (2023). Application
of Physical-Chemical Approaches for Encapsulation of Active Substances
in Pharmaceutical and Food Industries. Foods.

[11] Bamidele O. P., Emmambux M. N. (2021). Encapsulation of bioactive compounds
by “extrusion”
technologies: a review. Crit. Rev. Food Sci.
Nutr..

[12] Hong C. G., Florida E., Li H., Parel P. M., Mehta N. N., Sorokin A. V. (2023). Oxidized low-density
lipoprotein associates with cardiovascular
disease by a vicious cycle of atherosclerosis and inflammation: A
systematic review and meta-analysis. Front.
Cardiovasc. Med..

[13] Witztum J. L., Steinberg D. (2001). The Oxidative
Modification Hypothesis of Atherosclerosis:
Does It Hold for Humans?. Trends Cardiovasc
Med. [Internet]..

[14] Rabizadeh S., Seyedi S. A., Nabipoorashrafi S. A., Omidvar Siahkalmahalleh M., Yadegar A., Mohammadi F. (2023). The lack of association
between different LDL-C levels and oxidized LDL in patients with type
2 diabetes. Chronic Dis Transl Med. 1o de dezembro
de.

[15] Lin W. S., Leland J. V., Ho C. T., Pan M. H. (2020). Occurrence, Bioavailability,
Anti-inflammatory, and Anticancer Effects of Pterostilbene. J. Agric. Food Chem..

[16] Justino A. B., Franco R. R., Silva H. C. G., Saraiva A. L., Sousa R. M. F., Espindola F. S. (2019). B procyanidins of Annona crassiflora fruit peel inhibited
glycation, lipid peroxidation and protein-bound carbonyls, with protective
effects on glycated catalase. Sci. Rep..

[17] Benzie, I. F. F. ; Devaki, M. The ferric reducing/antioxidant power (FRAP) assay for non-enzymatic antioxidant capacity: concepts, procedures, limitations and applications. In Measurement of Antioxidant Activity & Capacity: Recent Trends and Applications; Apak, R. ; Capanoglu, E. ; Shahidi, F. , Wiley, 2017.

[18] Benzie I. F. F., Strain J. J. (1999). Ferric reducing/antioxidant power
assay: Direct measure
of total antioxidant activity of biological fluids and modified version
for simultaneous measurement of total antioxidant power and ascorbic
acid concentration. Em: Métodos em Enzimologia
[Internet]..

[19] Cao G., Alessio H. M., Cutler R. G. (1993). Oxygen-Radical Absorbance Capacity
Assay For Antioxidants. Free Radic Biol. Med..

[20] Brand-Williams W., Cuvelier M. E., Berset C. (1995). Use of a free
radical method to evaluate
antioxidant activity. LWT Food Sci. Technol..

[21] de
Lima Júnior J. P., Franco R. R., Saraiva A. L., Moraes I. B., Espindola F. S. (2021). Anacardium humile St. Hil as a novel source of antioxidant,
antiglycation and α-amylase inhibitors molecules with potential
for management of oxidative stress and diabetes. J. Ethnopharmacol..

[22] Chung B. H., Wilkinson T., Geer J. C., Segrest J. P. (1980). Preparative and
quantitative isolation of plasma lipoproteins: rapid, single discontinuous
density gradient ultracentrifugation in a vertical rotor. J. Lipid Res..

[23] Bradford M. M. (1976). A Rapid
and Sensitive Method for the Quantitation of Microgram Quantities
of Protein Utilizing the Principle of Protein-Dye Binding. Anal. Biochem..

[24] Gieseg S. P., Esterbauer H. (1994). Low density
lipoprotein is saturable by pro-oxidant
copper. FEBS Lett..

[25] Yagi K. (1998). Simple Assay
for the Level of Total Lipid Peroxides in Serum or Plasma. Em: Free Radical and Antioxidant Protocols..

[200] Raji M., Amad M.'a., Emwas A.-H. (2013). Dehydrodimerization
of pterostilbene during electrospray ionization mass spectrometry. Rapid Commun Mass Spectrom.

[26] Catenacci L., Vicatos A. I., Sorrenti M., Edmonds-Smith C., Bonferoni M. C., Caira M. R. (2023). Complexation between
the Antioxidant
Pterostilbene and Derivatized Cyclodextrins in the Solid State and
in Aqueous Solution. Pharmaceuticals.

[27] Mladenov M., Lubomirov L., Grisk O., Avtanski D., Mitrokhin V., Sazdova I. (2023). Oxidative Stress, Reductive Stress and Antioxidants
in Vascular Pathogenesis and Aging. Antioxidants.

[28] Bińkowska W., Szpicer A., Stelmasiak A., Wojtasik-Kalinowska I., Półtorak A. (2024). Microencapsulation of Polyphenols
and Their Application in Food Technology. Appl.
Sci..

[29] Rezagholizade-shirvan A., Soltani M., Shokri S., Radfar R., Arab M., Shamloo E. (2024). Bioactive compound
encapsulation: Characteristics,
applications in food systems, and implications for human health. Food Chem.: X.

[30] Yousefi M., Shadnoush M., Sohrabvandi S., Khorshidian N., Mortazavian A. M. (2021). Encapsulation
systems for delivery of flavonoids: A
review. Biointerface Res. Appl. Chem..

[31] Rahaman M. M., Hossain R., Herrera-Bravo J., Islam M. T., Atolani O., Adeyemi O. S. (2023). Natural
antioxidants from some fruits, seeds,
foods, natural products, and associated health benefits: An update. Food Sci. Nutr..

[32] Gonzales G.
B., Smagghe G., Grootaert C., Zotti M., Raes K., Camp J. V. (2015). Flavonoid
interactions during digestion, absorption,
distribution and metabolism: A sequential structure-activity/property
relationship-based approach in the study of bioavailability and bioactivity. Drug Metab. Rev..

[33] Syahputra R. A., Dalimunthe A., Utari Z. D., Halim P., Sukarno M. A., Zainalabidin S. (2024). Nanotechnology and flavonoids: Current research
and future perspectives on cardiovascular health. J. Funct. Foods.

[34] Frent O. D., Vicas L. G., Duteanu N., Morgovan C. M., Jurca T., Pallag A. (2022). Sodium
Alginate—Natural Microencapsulation Material
of Polymeric Microparticles. Int. J. Mol. Sci..

[35] Davidov-Pardo G, McClements D. J. (2014). Resveratrol encapsulation: Designing delivery systems
to overcome solubility, stability and bioavailability issues. Trends Food Sci. Technol..

[36] Steiner B. M., McClements D. J., Davidov-Pardo G. (2018). Encapsulation
systems for lutein:
A review. Trends Food Sci. Technol..

[37] Kumar S., Pandey A. K. (2013). Chemistry and biological activities
of flavonoids:
An overview. Sci. World J..

[38] Roy A., Khan A., Ahmad I., Alghamdi S., Rajab B. S., Babalghith A. O. (2022). Flavonoids a Bioactive Compound from Medicinal
Plants and Its Therapeutic Applications. BioMed
Res. Int..

[39] Martínez-Ballesta M., Gil-Izquierdo Á., García-Viguera C., Domínguez-Perles R. (2018). Nanoparticles
and controlled delivery for bioactive compounds: Outlining challenges
for new “smart-foods” for health. Foods.

[40] Gregor J. E., Fenton E., Brokenshire G., Van Den Brink P., O’Sullivan B. (1996). Interactions of calcium and aluminium ions with alginate. Water Res. [Internet]..

[41] Donati I., Christensen B. E. (2023). Alginate-metal
cation interactions: Macromolecular
approach. Carbohydr. Polym..

[42] Lee K. Y., Mooney D. J. (2012). Alginate: Properties and biomedical
applications. Prog. Polym. Sci..

[43] Kumar, A. ; Kothari, A. ; Kumar, P. ; Singh, A. ; Tripathi, K. ; Gairolla, J. Introduction to Alginate: Biocompatible, Biodegradable, Antimicrobial Nature and Various Applications. In Alginate-Applications and Future Perspectives, IntechOpen, 2023. www.intechopen.com.

[44] Prior R. L. (2015). Oxygen
radical absorbance capacity (ORAC): New horizons in relating dietary
antioxidants/bioactives and health benefits. J. Funct. Foods.

[45] Zhang L., Zhou G. Z., Song W., Tan X. R., Guo Y. Q., Zhou B. (2012). Pterostilbene
protects vascular endothelial cells against
oxidized low-density lipoprotein-induced apoptosis in vitro and in
vivo. Apoptosis. janeiro de.

[46] Zhang Y., Zhang Y. (2016). Pterostilbene, a novel
natural plant conduct, inhibits high fat-induced
atherosclerosis inflammation via NF-κB signaling pathway in
Toll-like receptor 5 (TLR5) deficient mice. Biomedicine and Pharmacotherapy. 1o de julho de.

[47] Park E. S., Lim Y., Hong J. T., Yoo H. S., Lee C. K., Pyo M. Y. (2010). Pterostilbene,
a natural dimethylated analog of resveratrol, inhibits
rat aortic vascular smooth muscle cell proliferation by blocking Akt-dependent
pathway. Vascul Pharmacol. julho de.

[48] Chen Y., He W., Qiu J., Luo Y., Jiang C., Zhao F. (2024). Pterostilbene improves neurological dysfunction and neuroinflammation
after ischaemic stroke via HDAC3/Nrf1-mediated microglial activation. Cell Mol. Biol. Lett..

[49] Wu X., Kang J., Xie C., Burris R., Ferguson M. E., Badger T. M. (2010). Dietary
blueberries attenuate atherosclerosis
in apolipoprotein E-deficient mice by upregulating antioxidant enzyme
expression. Journal of Nutrition. setembro de.

[50] Liu Q., Chen J., Qin Y., Jiang B., Zhang T. (2020). Zein/fucoidan-based
composite nanoparticles for the encapsulation of pterostilbene: Preparation,
characterization, physicochemical stability, and formation mechanism. Int. J. Biol. Macromol..

[51] Yang G., Sun J., Lu K., Shan S., Li S., Sun C. (2021). Pterostilbene
Coupled with Physical Exercise Effectively Mitigates Collagen-Induced
Articular Synovial by Correcting the PI3K/Akt/NF-κB Signal Pathway. J. Agric. Food Chem..

[52] Lacerda D., Türck P., Campos-Carraro C., Hickmann A., Ortiz V., Bianchi S. (2020). Pterostilbene improves cardiac function in a rat model
of right heart failure through modulation of calcium handling proteins
and oxidative stress. Appl. Physiol., Nutr.,
Metab..

[53] Kosuru R., Kandula V., Rai U., Prakash S., Xia Z., Singh S. (2018). Pterostilbene Decreases Cardiac Oxidative Stress and
Inflammation
via Activation of AMPK/Nrf2/HO-1 Pathway in Fructose-Fed Diabetic
Rats. Cardiovasc Drugs Ther..

[54] Barboza
Duarte Rodrigues I. F., Pereira J. M., de Lima L. M. S., Lins
Silva K. G., Silva M. R., da Costa Silva V. (2025). Development and evaluation of capsules loaded with red propolis extract
and metallic nanoparticles using the ionic gelation method. J. Apic. Res..

[55] Ramos P. E., Cerqueira M. A., Teixeira J. A., Vicente A. A. (2018). Physiological protection
of probiotic microcapsules by coatings. Crit
Rev. Food Sci. Nutr..

[56] Sood A., Dev A., Das S. S., Kim H. J., Kumar A., Thakur V. K., Han S. S. (2023). Curcumin-loaded
alginate hydrogels for cancer therapy
and wound healing applications: A review. Int.
J. Biol. Macromol..

[57] Conte R., De Luca I., Valentino A., Cerruti P., Pedram P., Cabrera-Barjas G., Moeini A., Calarco A. (2023). Hyaluronic Acid Hydrogel
Containing Resveratrol-Loaded Chitosan Nanoparticles as an Adjuvant
in Atopic Dermatitis Treatment. J. Funct. Biomater..

[58] Jin M., Li S., Wu Y., Li D., Han Y. (2021). Construction of Chitosan/Alginate
Nano-Drug Delivery System for Improving Dextran Sodium Sulfate-Induced
Colitis in Mice. Nanomaterials (Basel)..

[59] Manjili Z. N., Mahoonak A. S., Ghorbani M., Tabarestani H. S., Moghadam V. E. (2024). Composite alginate-based hydrogel
delivery of antioxidant
pumpkin protein hydrolysate in simulated gastrointestinal condition. Curr. Res. Food Sci..

[60] Xu, Z. , Lam, M. T. Alginate Application for Heart and Cardiovascular Diseases. In Alginates and Their Biomedical Applications, Rehm, B. , Moradali, M. (eds), Springer Series in Biomaterials Science and Engineering, vol 11, Springer: Singapore, 2018. 10.1007/978-981-10-6910-9_7.

[61] Kumar A., Belhaj M., DiPette D. J., Potts J. D. (2021). A Novel Alginate-Based
Delivery System for the Prevention and Treatment of Pressure-Overload
Induced Heart Failure. Front Pharmacol..

