# Supplementary material for: Encapsulated Pterostilbene in pH-Sensitive Alginate Beads for LDL Oxidation Inhibition and Antioxidant Protection
Source: ACS Omega. 2026 Mar 17;11(12):18642–52. doi: 10.1021/acsomega.5c07990 (PMC13044650; doi:10.1021/acsomega.5c07990)
Supplement: Supplementary file 1 [file ao5c07990_si_001.pdf]

Encapsulated Pterostilbene in pH-Sensitive Alginate Beads for LDL Oxidation Inhibition and Antioxidant Protection

Rener Mateus Francisco Duarte[1-3*], Jéssica Maria Pereira[1], Livia Maria Santos de Lima[1], Tarcísio Paiva Mendonça[2], Vinicius Prado Bittar[2], Maria Sol Peña Carrillo[2], Jeniffer Mclaine Duarte de Freitas[5], Ilza Fernanda Barboza Duarte Rodrigues[1,3], Johnnatan Duarte de Freitas[5], Irinaldo Diniz Basílio Júnior[4], Allisson Benatti Justino[2], Foued Salmen Espindola[2], Anielle Christine Almeida Silva[1*]

1- Strategic Materials Laboratory, Institute of Physics, Federal University of Alagoas, Maceió, AL, Brazil
2- Biochemistry and Molecular Biology Laboratory, Institute of Biotechnology, Federal University of Uberlandia, Uberlandia, Brazil.
3- Northeast Biotechnology Network (RENORBIO), Federal University of Alagoas, Maceio, Brazil.
4- Drug Technology and Control Laboratory, Institute of Pharmaceutical Sciences, Federal University of Alagoas, Maceio, Brazil.
5- Laboratory of Instrumental Analysis, Federal Institute of Alagoas, Maceio, Brazil.

*Corresponding author:

Anielle Christine Almeida Silva (aniellechristineas@gmail.com), Institute of Physics, Federal University of Alagoas, Maceió, AL, Brazil

Rener Mateus Francisco Duarte (renermateus3@gmail.com), Institute of Chemistry, Federal University of Alagoas, Maceió, AL, Brazil

Supporting Information

A

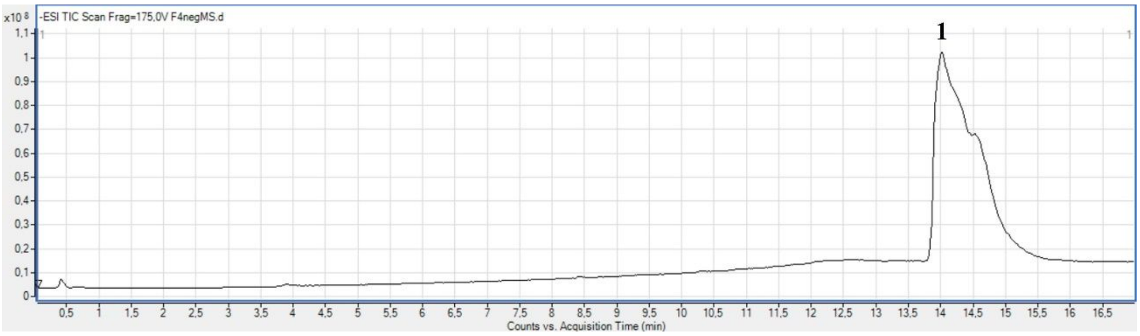

B

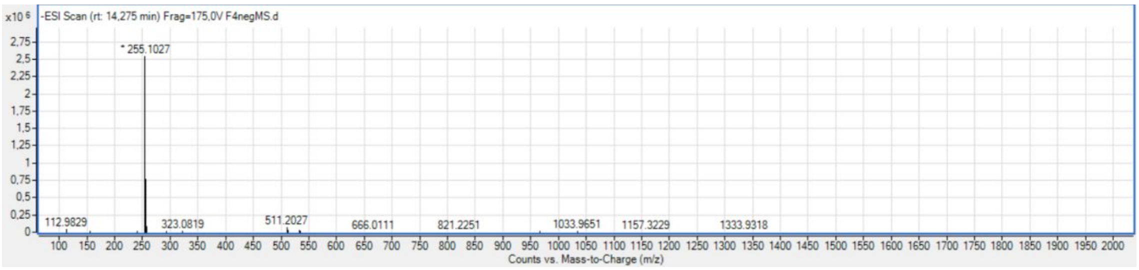

Figure S1. Chromatograms of pterostilbene extract samples by HPLC-ESI-MS/MS. (A) Chromatograms of pterostilbene extract samples by HPLC-ESI-MS/MS (negative mode) and (B) Chromatogram of pterostilbene from pterostilbene extract sample by HPLC-ESI-MS/MS (*m*/*z* 255 $[M-H]^-$).

Table S1. Physicochemical Parameters and Encapsulation Characteristics of Alginate–Pterostilbene Microbeads

Parameter	Value	Description
Mean diameter (cm)	0.30 ± 0.02	Measured by digital caliper from ≥ 10 beads.
Estimated volume (μL)	14 ± 2	Calculated assuming spherical geometry ($V = 4/3\pi r^3$).
Initial pterostilbene concentration ($mg\ mL^{-1}$)	31.25	Concentration on precursor polymer solution before gelation.
Theoretical drug loading (mg per bead)	0.44	Calculated from the mean bead volume × initial drug concentration.
Directly measured drug loading (mg per bead)	0.55 ± 0.06	Determined after dissolving individual beads and quantifying released pterostilbene.
Encapsulation efficiency (indirect method, %)	> 90%	Based on unencapsulated drug in the supernatant after gelation.
